# On Goitre in Switzerland

**Published:** 1845-12

**Authors:** F. H. Hamilton


*On Goitre in Switzerland, Extract from "Notes of an European Tour.*" By Professor F. H. Hamilton.—I think no one can travel through this Canton, (Valais,) without being convinced that, whatever causes may produce goitre elsewhere, here at least it has its source in poverty, filth, indolence, a meagre, coarse diet, humid air and exclusion from light; causes which, from the peculiar disinclination of the inhabitants to migrate or marry beyond their own Canton, have, by ceaseless inheritance, been accumulating in intensity through a long succession of generations; for the children of goitrous parents and grand parents are generally *born* goitrous, and the intermarriage of persons born goitrous produces a cretin offspring. And we may account for the greater prevalence of goitre here than perhaps in any other part of Europe, from the more perfect combination of the causes enumerated, especially exclusion from pure air and light, the valley being the deepest in Europe, and that portion of it which extends between St. Maurice and St. Martigny being very narrow, and extending directly north and south, is visited by the sun only a few hours of the day; while the parallel mountains which verge the river, and the lofty barrier of Dent du Midi and Dent de Morcles on the north, and St. Bernard on the south, effectually cut off the wind from whatever point it may blow. Here then is a basin of stagnant air which has never been stirred since the mountains took their present form, and which must for ever remain, despite what art or civilization may do, given up to this horrible disease.

Other causes have been assigned for the existence of goitre and cretinism along this valley; none of them, however, will bear examination. The "custom of carrying burdens upon the head" so universal here, is no less universal among the peasantry in nearly all parts of Europe, and if it was adequate to the production of goitre here, it would prove equally adequate elsewhere. The "habitual use of snow water" by the Valaisans cannot be denied, since not only the Rhone itself, but all its small tributaries and even most of the springs, are supplied directly by the glaciers which are for ever above them. Yet how much this has to do with the production of goitre is understood when it is known that a removal to the summits of the mountains where snow water *must* be the sole drink, is the most certain cure of both goitre and cretinism, and that if the transplantation occur sufficiently early it will often effectually prevent their development. Nor can I ascribe any more weight to the large amount of "calcareous, magnesian and silicious particles" which those who drink the waters of the Rhone necessarily receive, and which constantly render the river turbid, and cover its banks with a white sand, since neither lime, magnesia, nor silex have yet been shown to produce goitre. I know many districts in which lime is the only rock and the waters are highly impregnated, and yet the malady in question does not prevail—the same, it is not denied, may be said of many primitive regions. I attach also great importance to the argument that if foreign particles suspended in the water was actually the cause, it would be so easily ascertained, that it could not fail of having been known long since, by confining, either accidentally or intentionally, persons still residing in the valley to snow artificially melted and deprived of all foreign elements. The proof would be so natural and easy that, I say, it *must* have been made and the doubt resolved.

You remember my suddenly-formed opinion, when I saw both goitre and scrofula at Geneva, that both sprang from the same or similar sources, which opinion I declared I would hold with the privilege of changing if I found occasion hereafter. The opinion is confirmed. I do not say positively that the diseases are the same, but certainly they have like causes and many other points in common. Scrofula is hereditary, so is goitre; goitre occurs oftenist in children of light complexion, flaxen hair, large blue eyes, soft, relaxed fibres, and who present not frequently a precocity of intellect; the same is true of scrofula. Valaisans have what we term a scrofulous look. Many are ricketty or otherwise deformed, yet seldom did I see scrofulous enlargement in the absorbant glands of the neck, and, as I believe, because the existence of a tumor often as large as the head and sometimes twice as large, (Dr. Mott says he saw at Martigny one of such magnitude that " the poor woman was obliged to crawl along the floor upon her hands and feet dragging the gigantic dewlap and pendulous mass after her,") serves as a sufficient drain and prevents their development—precisely as the existence of one large scrofulous tumor retards the growth of others, while its removal is followed often by a numerous crop of new sprouts along the whole chain of absorbents. I am aware that *in Scotland scrofula is common and goitre is rare*, but having travelled some among the peasantry of the Highlands and the adjacent valleys, and observed their habits, modes of living, climate, I am convinced that, to say the least, we are not entitled to the inference drawn by Dr. Gibson, that *therefore* scrofula and goitre cannot be confounded.—Precisely the causes which produce goitre in Switzerland, fair complexion, filthy cabins, meagre, coarse vegetable diet, exclusion from air and light, exist also in the valleys of the Scotch Highlands, only ina less degree of intensity—and the only inference which could be legitimately made would be that *these causes operating moderately produce scrofula, and operating more intensely produce goitre.*

Farther, the diseases might seem to have a common source, since they are subject to common remedies. Not to be more particular, I may mention the use of iodine, which is regarded as almost equally a specific for both,—tonics, cleanliness, nutritious and wholesome diet, and also removal into a more clear and invigorating air. I might also add that, while exclusion from light alone has been proven by experiment to produce in dogs " softening of the bones and rickets," (a scrofulous constitution;) so here the goitre extends to dogs, goats, sheep, cattle, horses, &c.—*Buffalo Med. Jour.*

